# Attitude Towards Deposit Limits and Relationship with Their Account-Based Data Among a Sample of German Online Slots Players

**DOI:** 10.1007/s10899-022-10155-1

**Published:** 2022-08-24

**Authors:** Michael Auer, Mark D. Griffiths

**Affiliations:** 1 Neccton Ltd, Muhlgasse 23, 9900 Lienz, Austria; 2 International Gaming Research Unit, Psychology Department, Nottingham Trent University, 50 Shakespeare Street, NG1 4FQ Nottingham, UK

**Keywords:** Responsible gambling, Problem gambling, Limit-setting, Behavioral tracking data, Survey data

## Abstract

Social responsibility and duty of care have become major cornerstones for gambling operators. This has led to the introduction of many different responsible gambling tools such as limit-setting, mandatory play breaks, and personalized messaging. In the present study, the authors were given access to two secondary datasets provided by a German online slots game operator. The first dataset was from an online survey carried out by the gambling operator among 1000 of its players concerning their attitude towards deposit limits as well as self-reported problem gambling. In addition to the survey responses, the authors were given access to a second dataset of account-based data concerning each customer’s wagers, wins, monetary deposits, and monetary withdrawals. These datasets were then combined. The majority of players had a positive attitude towards the maximum deposit monthly deposit limit which was introduced by the German State Treaty on Gambling in 2021. Players who disagreed with the maximum monthly deposit limit, deposited significantly more money in the 30 days prior to answering the survey questions compared to players who agreed with the monthly deposit limit. The tracking data found only 7.6% of players had deposited the maximum amount of money allowed in one month. However, 60.5% of players in the survey data said that they did so. Players who said that they continued to gamble after reaching the deposit limit wagered and deposited significantly more money in the 30 days prior to the survey compared to players who said they stopped gambling after reaching the deposit limit. Two-fifths of players said they continued to gamble after reaching the monthly deposit limit (42%). The majority of the players said they chose a personal deposit limit because it helped them to better control their gambling expenditure. A quarter of the players reported gambling problems using the Brief Biosocial Gambling Screen (27%). Self-reported problem gambling was not correlated with depositing, wagering or any other player tracking metric.

## Introduction

In recent years many gambling operators have identified social responsibility and duty of care as a major cornerstone of their operations (Harris & Griffiths, 2017). Gambling companies can offer players a number of tools and procedures that help minimize gambling-related harm. Among these are voluntary limit-setting (Auer & Griffiths, 2013) and voluntary self-exclusion (Motka et al., 2018). Catania and Griffiths (2021) analyzed 50 popular online operators with respect to their responsible gambling (RG) practices. They found that 49 operators offered RG tools such as limit-setting tools and short periods of taking a break from gambling. Almost all operators offered self-exclusion tools (49 out of 50).

In relation to voluntary limit-setting, players can (or have to) choose the maximum amount they are willing to wager, lose or deposit over different periods of time (e.g., session, day, week or month). Typically, players cannot immediately increase a limit but decreasing a limit usually comes into effect instantly. Often, online gambling sites also offer players the opportunity to limit the time spent gambling for different time periods. Most European online gambling regulations (e.g., Spain, UK, Germany, Sweden, Denmark, Switzerland, Austria, Norway) specifically require operators to provide voluntary limit-setting tools to players. Players typically have to choose a limit, but the size of the limit is voluntary.

Voluntary limit-setting can be seen as a pre-commitment strategy (Williams, 2010). Ladouceur et al. (2012) specified pre-commitment as a system which enables gamblers to set limits on money and time expenditure prior to the commencement of a gambling session. It is based on the principle that players should make decisions about their gambling in a non-aroused state. Research has shown that players can experience high levels of arousal (Wilkes, Gonsalvez, & Blaszczynski, 2010) and dissociative states (Rogier et al., 2021) while gambling. These states can prevent players from being able to stop gambling (Delfabbro & Winefield, 1999; Dowling et al., 2005; Petry, 2005).

Several studies have analyzed the impact of voluntary limit-setting in ecologically valid settings with real-world players. Auer and Griffiths (2013) analyzed the impact of voluntary deposit limits and time limits on subsequent gambling behavior among 5000 Austrian online gamblers. Among the 10% most intense players (measured by theoretical loss which is the amount of money wagered multiplied by the probability of winning), they found that the setting of voluntary deposit limits had the highest significant effect on subsequent monetary spending among casino and lottery gamblers. Monetary spending among poker players significantly decreased after setting a voluntary time limit. Similarly, Auer, Hopfgartner and Griffiths (2020) analyzed the impact of voluntary limit-setting behavior on subsequent spending among 49,560 players of the online gambling operator *Kindred*. Players who had voluntarily set a monetary limit spent significantly less than players who did not set a limit one year after.

However, some researchers have raised doubts about the efficacy of voluntary limit-setting. Three systematic reviews failed to find clear evidence of the effectiveness of monetary pre-commitment (Ladouceur et al., 2012, 2017; Drawson et al., 2017). Other studies using account-based tracking data have reported that the uptake of voluntary limit-setting is low. For instance, Auer et al. (2020) reported only 1.31% of online gamblers at *Kindred* voluntarily set a limit during a three-month period, and another study by the same authors reported that only 8% of gamblers at *Kindred* voluntarily set a limit during a six-months period (Auer, Hopfgartner & Griffiths, 2021).

Ivanova et al. (2019) conducted a randomized trial with the Finnish online gambling operator *PAF*. They prompted players to set a deposit limit of optional size either (i) at registration, (ii) before or (iii) after their first deposit, or (iv) to an unprompted control condition. They did not find any differences between the groups with respect to net loss. Players who were prompted to set a limit did so more frequently compared to the control condition. They concluded that prompting online gamblers to set a voluntary deposit limit of optional size did not affect subsequent net loss compared to unprompted customers. It was suggested that the design of pop-up messages should be improved and that other pre-commitment tools should be further evaluated.

Some countries and selected operators have introduced mandatory deposit limits or loss limits. In Norway, players cannot lose more than NOK 20,000 (approximately $2,000 US) with the government-owned gambling operator *Norsk Tipping* (Auer, Reiestad & Griffiths, 2020). In Austria, players of the only licensed online casino *win2day* cannot deposit more than €800 per week (Auer & Griffiths, 2013) which is still the case at the time of writing. In 2021, Germany introduced a new state treaty on gambling which forces gambling operators to set a maximum deposit limit of €1000 per month across all licensed operators. Sweden introduced a SEK 5000 (approximately $500 US) maximum weekly deposit limit in June 2020 as a response to the global COVID-19 pandemic (Auer & Griffiths, 2021).

Ecologically valid research with real-world players concerning mandatory limit-setting has only been conducted in Norway. Using account-based tracking data, Auer et al. (2018) studied the effect of personalized feedback on subsequent gambling behavior for players who had reached 80% of their personal monthly loss limit at the *Norsk Tipping* online gambling website. Using a matched-pairs design, results showed that those gamblers receiving personalized feedback in relation to limit-setting showed a significant reduction in the amount of money gambled.

In an online survey, Auer, Reiestad and Griffiths (2020) asked 2352 online gamblers with the Norwegian gambling operator *Norsk Tipping* about their attitude to the then recently introduced monthly global loss limit. They found that three-quarters of the sample were aware the new global loss limit, two-thirds of the sample knew how to set limits on their gambling, and four-fifths of the sample had a positive attitude towards the global loss limit. Very few gamblers played with other operators after they had reached their spending limits. They concluded that the introduction of a global loss limit had a positive impact among *Norsk Tipping’s* clientele.

Delfabbro and King (2021) conducted a literature review regarding the efficacy of voluntary and mandatory limit-setting. Based on the findings, they concluded that voluntary pre-commitment systems may be better used as a tool in conjunction with other responsible gambling features (e.g., in combination with voluntary self-exclusion schemes). They also noted that the Norwegian experience would appear to indicate that mandatory limit-setting or pre-commitment schemes might be the only effective way to reduce the harms associated with excessive gambling. However, they also noted that higher-risk gamblers might choose to gamble elsewhere with less-regulated or unregulated gambling operators if they are forced to set limits.

The present study investigated the attitude towards limit-setting and more specifically the monthly maximum deposit limit of €1,000 which was introduced by the German State Treaty on Gambling in 2021. The study was conducted with real-world players from a German online gambling site. The German online gambling environment is the first regulation which enforces a maximum deposit limit across all operators licensed in a country. However, this could also lead to undesired effects if players increasingly migrate to less regulated online gambling sites. Only one previous study reported attitudes towards maximum spending limits (i.e., Auer et al., 2020). Therefore, the authors believe that the present study could significantly contribute to the understanding of player behavior related to limit-setting. The study was purposefully exploratory in nature and for that reason there were no specific hypotheses.

## Method

### Contextual Background: German Online Gambling Regulation

In September 2021, Germany introduced a new state treaty on gambling. The German State Treaty specifies three online gambling licenses:

- *Virtual slots*: Under this license, only slots can be offered.
- *Table and card games*: Under this license, table games such as roulette, blackjack, and baccarat can be offered.
- *Casino games*: Under this license, casino games with live dealers can be offered.

Sports betting operators need to hold a different license. Lottery licenses are issued by the German Bundesländer. Operators can offer any combination of the three aforementioned online gambling licenses, including sports betting. The three aforementioned online gambling licenses are all subject to a monthly deposit limit of €1000 per player. Each licensee is connected to a governmental database in order to ensure that a single player cannot deposit more than €1000 in one month across all licensed gambling operators in Germany. There are a number of other restrictions such as a maximum wager per game, a minimum time span between two games, and a mandatory pause when a player switches between virtual slots, table/card games, and casino games. Apart from the €1,000 monthly maximum deposit limit, it is mandatory for players have to choose a personal monthly deposit limit during registration.

At the time of writing, the German state had not yet issued a single online gambling license. However, the gambling operator which provided the data was compliant with the German State Treaty and players could not deposit more than €1000 per month. The operator only offered slot games. However, players could also gamble with other compliant (as well as non-compliant) operators.

### Participants

The present authors were given access to two secondary datasets. The first dataset was from an online survey carried out by the gambling operator among its clientele. In mid-February 2022, a German online slots game operator collected responses from 1000 customers about their attitude towards deposit limits as well as self-reported problem gambling. Players were asked to answer the survey after logging into their account. Only players who had registered for at least 30 days before were asked to answer the survey. The mean average age was 40 years (SD = 10) and 18% of the participants were female (n = 180). In addition to the survey responses, the authors were given access to a second dataset of account-based data concerning each customer’s wagers, wins, monetary deposits, and monetary withdrawals, as well as the exact timestamp of the single transactions since the online slots game operator launched in June 1, 2021.

### Measures

The survey comprised a number of items regarding players’ attitudes towards limits, their reactions when reaching limits, their reasons for setting limits, and the three items of the Brief Biosocial Gambling Screen (BBGS; Gebauer et al., 2010). The four questions regarding the attitude towards limits were: (i) *“I feel positive towards the €1000 maximum deposit limit”*, (ii) *“I believe that a maximum deposit limit is relevant for me”*, (iii) *“I believe that generally I have a sufficient overview of my gambling expenditures”*, and (iv) *“Deposit limits help me to maintain a sufficient overview of, and control over, how much money I lose”.* These were responded to as either ‘Disagree entirely’, ‘Disagree in part’, ‘Neither agree nor disagree’, ‘Agree in part’, ‘Agree entirely’, and ‘Don't know’.

There were also five questions concerning the reaching of limits that could be answered ‘yes’ or ‘no’: (i) *“I have never reached the maximum deposit limit”*, (ii) *“I did not play for the rest of the month”*, (iii) *“I did not play until I was permitted again”*, (iv) *“I continued to play with other compliant operators”* (the criteria for compliance are €1 maximum bet, minimum 5-second spin, and €1000 deposit limit), and (v) *“I continued to play with other non-compliant online operators”.* Additionally, there were three options concerning reasons for setting limits that could be answered ‘yes’ or ‘no’: (i) *“I set a personal deposit limit because I have to”*, (ii) *“I set a personal deposit limit to better control my gambling expenditure”*, and (iii) *“I don't know why I set a deposit limit”.*

Finally, the three questions of the BBGS could also be answered ‘yes’ or ‘no’: (i) *“During the past 12 months, have you become restless, irritable or anxious when trying to stop/cut down on gambling?”*, (ii) *“During the past 12 months, have you tried to keep your family or friends from knowing how much you gambled?”*, and (iii) *“During the past 12 months did you have such financial trouble as a result of your gambling that you had to get help with living expenses from family, friends or welfare?”.*

### Statistical Analysis

Kruskal-Wallis tests were used to test the differences of metric variables across groups of players (Mahoney et al., 1996). A logistic regression was applied to predict self-reported problem gambling with the survey variables as well as player tracking data as independent variables. The authors tested whether the amount of money deposited and wagered followed a normal distribution according to D’Agostino and Rosman (1971). The analysis program *Python* (Pedregosa et al., 2011) was used for the data analysis.

## Results

**Fig. 1:**
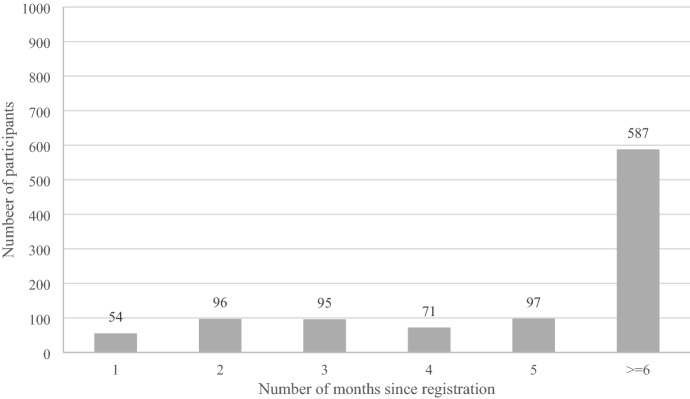
Distribution of the number of months since registration among players (N = 1000)

The amount of money deposited (K^2^ = 578, *p* < 0.001) and the amount wagered (K^2^ = 700, *p* < 0.001) in the 30 days prior to answering the survey significantly deviated from a normal distribution. Figure 1 shows the number of players who were registered for one to five months and six or more months. Nearly three-fifths of the 1000 players had been registered for six or more months (58.7%).

A series of questions were asked in order to understand players attitudes towards the maximum deposit limit and deposit limits in general (Tables 1 and 2). Asked whether they felt positive towards the €1000 monthly maximum deposit limit, 62.4% of players agreed in part or entirely, 21% of players disagreed entirely or in part. Moreover, over one-third of the players agreed in part or entirely that a maximum deposit limit was relevant for them (36%). The same percentage of players disagreed in part or entirely that a maximum deposit limit was relevant for them (36%).

**Table 1:** Attitude towards the maximum deposit limit among players (N = 1000)

	I feel positive towards the €1000 maximum deposit limit	I believe that a maximum deposit limit is relevant for me
Disagree entirely	136 (13.6%)	245 (24.5%)
Disagree in part	71 (7.1%)	112 (11.2%)
Neither	169 (16.9%)	224 (22.4%)
Agree in part	100 (10.0%)	103 (10.3%)
Agree entirely	524 (52.4%)	257 (25.7%)
Don't know	0 (0%)	59 (5.9%)

**Table 2:** Personal relevance of deposit limits among players (N = 1000)

	I believe that generally I have a sufficient overview of my gambling expenditure	Deposit limits help me to maintain a sufficient overview of, and control over, how much money I lose
Disagree entirely	43 (4.3%)	142 (14.2%)
Disagree in part	50 (5.0%)	70 (7.0%)
Neither	174 (17.4%)	205 (20.5%)
Agree in part	177 (17.7%)	124 (12.4%)
Agree entirely	473 (47.3%)	353 (35.3%)
Don't know	83 (8.3%)	106 (10.6%)

Two-thirds of players agreed entirely or in part when asked whether they believed they had a sufficient overview of their gambling expenditure (65%). Approximately one-tenth of players disagreed entirely or in part when asked whether they believed they had a sufficient overview of their gambling expenditure (9.3%). Just under half of the players agreed entirely or in part when asked whether deposit limits were helpful to maintain a sufficient overview of, and control over, how much money they lose (47.7%). Approximately two-fifths disagreed entirely or in part when asked whether deposit limits were helpful to maintain a sufficient overview of, and control over, how much money they lose (21.2%).

Apart from the survey data, the authors also had access to the players’ account-based tracking data (e.g., actual wagering and depositing data). Out of the 1,000 participants, 76 had deposited the maximum amount of €1,000 in at least one month since they registered (7.6%). The mean average age of the 76 players who reached the maximum deposit limit in at least one month was 46 years (SD = 10). The mean average age of the players who did not reach the maximum deposit limit in any month since registration was 39 years (SD = 10). The difference was significant (*t* = 4.85, *p* < 0.001).

A chi-square test showed there was a significant difference between the number of females and males with respect to reaching the limit (χ^2^ = 6.89, *p* = 0.0057, df = 1). More specifically, of the 76 players who reached the limit, 22 were female (29%). Of the 924 players who did not reach the limit, 158 were female (17%). Of the 76 players who reached the limit, 54 were male (71%). Of the 924 players who did not reach the limit, 766 were male (83%). Therefore, the percentage of females was significantly larger among the players who reached the limit compared to players who did not reach the limit.

Although the tracking data found only 76 players deposited €1,000 in at least one month, 605 players self-reported that they had reached the maximum deposit limit (60.5%). Of these 605 players, 253 said they continued to gamble (42%). Of these 253 players, 51 said they continued to gamble with other online operators who were compliant with the German regulation (20%), 48 said they continued to gamble with other online operators who were not compliant with the German regulation (19%), and 15 said they continued to gamble with land-based operators (6%). Nearly three-quarters of players answered two questions about the reasons to choose a personal deposit limit (73%). Of these, 172 said they chose a personal deposit limit because they had to (24%) and 394 said they chose a personal deposit limit because it helped them to better control their gambling expenditure (54%).

**Fig. 2:**
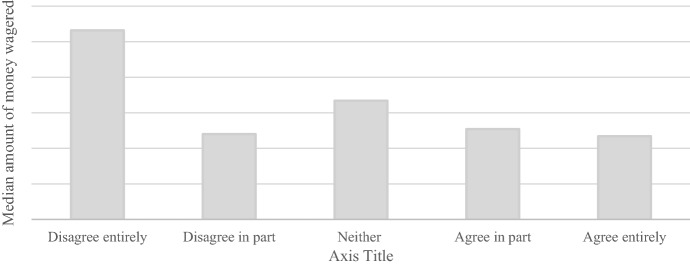
Median amount of money wagered in the thirty days prior to answering the survey per answer category for the question *“I feel positive towards the €1000 maximum deposit limit”*. N.B. Due to issues of commercial sensitivity, actual amount of money wagered is not shown

Players who disagreed entirely with the maximum deposit limit had the largest median amount of money wagered in the 30 days prior to answering the survey (Fig. 2). However, a Kruskal-Wallis test reported no significant difference between players who disagreed entirely or partly and players who agreed in part or entirely (K^2^ = 3.56, *p* = 0.056). Players who disagreed with the maximum deposit limit also had the largest median amount of money deposited in the 30 days prior to answering the survey. A Kruskal-Wallis test found a significant difference between players who disagreed entirely or partly and players who agreed in part or entirely (K^2^ = 7.96, *p* = 0.004).

**Fig. 3:**
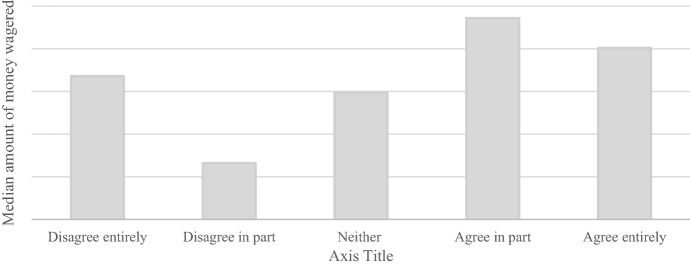
Median amount of money wagered in the thirty days prior to answering the survey per answer category for the question *“I believe that a maximum deposit limit is relevant for me”*. N.B. Due to issues of commercial sensitivity, actual amount of money wagered is not shown

Figure 3 shows the median amount of money wagered 30 days prior to answering the survey for each answer to the question *“I believe that a maximum deposit limit is relevant for me”*. Players who agreed in part or entirely that a maximum limit was relevant to them had the largest median amount of money wagered. Neither the amount of money deposited (K^2^ = 1.82, *p* = 0.18) nor the amount of money wagered in the 30 days prior to answering the survey were significantly different between players who disagreed entirely or in part and players who agreed in part or entirely (K^2^ = 1.88, *p* = 0.17).

**Fig. 4:**
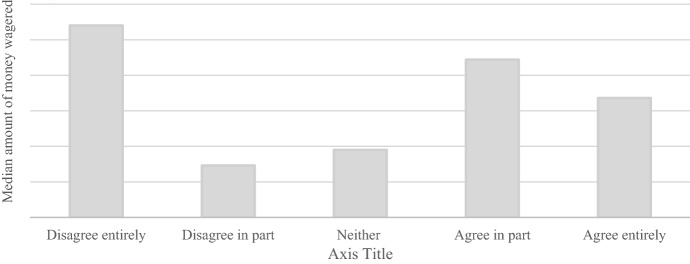
Median amount of money wagered in the thirty days prior to answering the survey per answer category for the question *“I believe that generally I have a sufficient overview of my gambling expenditure”*. N.B. Due to issues of commercial sensitivity, actual amount of money wagered is not shown

Figure 4 shows the median amount of money wagered 30 days prior to answering the survey for each answer to the question *“I believe that generally I have a sufficient overview of my gambling expenditure”.* Players who disagreed entirely to having had a sufficient overview of their gambling expenditure had the largest median amount of money wagered. Neither the amount deposited (K^2^ = 0.01, *p* = 0.97) nor the amount wagered (K^2^ = 0.009, *p* = 0.92) in the 30 days prior to answering the survey were significantly different between players who disagreed entirely or in part and players who agreed in part or entirely.

**Fig. 5:**
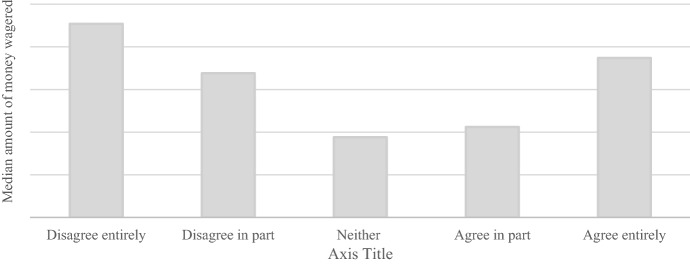
Median amount of money wagered in the thirty days prior to answering the survey per answer category for the question *“Deposit limits help me to maintain a sufficient overview of, and control over, how much money I lose”*. N.B. Due to issues of commercial sensitivity, actual amount of money wagered is not shown

Figure 5 shows the median amount of money wagered 30 days prior to answering the survey for each answer to the question *“Deposit limits help me to maintain a sufficient overview of, and control over, how much money I lose”.* Players who disagreed entirely that a deposit limit helped them had the largest median wager. Neither the amount of money deposited (K^2^ = 3.47, *p* = 0.06) nor the amount of money wagered (K^2^ = 2.88, *p* = 0.09) in the 30 days prior to answering the survey were significantly different between players who disagreed entirely or in part and players who agreed in part or entirely.

The 605 players who said that they continued to gamble after reaching the deposit limit, deposited and wagered more in the 30 days prior to answering the survey compared to players who said that they did not continue to gamble. Kruskal-Wallis tests found a significant difference with respect to the amount of money deposited (K^2^ = 26, *p* < 0.001) as well as the amount of money wagered (K^2^ = 17.32, *p* < 0.001). The 76 players who actually reached the €1000 in at least one month also deposited and wagered more in the 30 days prior to answering the survey questions compared to the players who never reached the monthly maximum monetary deposit limit. Kruskal-Wallis tests found a significant difference with respect to amount of money deposited (K^2^ = 131, *p* < 0.001) as well as amount of money wagered (K^2^ = 116, *p* < 0.001).

The 172 players who said they chose a personal deposit limit because they had to, deposited and wagered less money in the 30 days prior to answering the survey compared to the rest of the players did not agree to that statement. Kruskal-Wallis tests found no significant difference with respect to amount of money deposited (K^2^ = 3.41, *p* < 0.06) as well as amount of money wagered (K^2^ = 1.27, *p* < 0.26). The 394 players who said they chose a personal deposit limit because it helped them to better control their gambling expenditure, deposited and wagered more money in the 30 days prior to answering the survey compared to the rest of the players who did not agree to that statement. Kruskal-Wallis tests found a significant difference with respect to amount deposited (K^2^ = 14.9, *p* < 0.001) as well as amount wagered (K^2^ = 16.2, *p* < 0.001).

The survey also contained the three questions of the Brief Biosocial Gambling Screen. Out of the 1,000 players, 815 players answered the three items. Findings indicated that (i) approximately four-fifths of players said that during the past 12 months they had become restless, irritable or anxious when trying to stop/cut down on gambling (81.5%); (ii) approximately one-eighth of players said that during the past 12 months they had tried to keep their family or friends from knowing how much they gambled (13.3%), and (iii) approximately one-seventh of players said that during the past 12 months they had such financial trouble as a result of your gambling that they had to get help with living expenses from family, friends or welfare (14.04%).

In total 178 players agreed to at least one of the three statements and were regarded as problem gamblers (21.2%). In order to validate the answers provided, the authors carried out a logistic regression. Self-reported problem gambling was used as dependent variable and the remaining survey responses as well as the players’ actual gambling behavior in the past 30 days were used as independent variables. The logistic regression had a Nagelkerke R of 13.5%. The likelihood of being a problem gambler was larger for players who self-reported that they (i) had set a deposit limit was relevant for them, (ii) had an insufficient overview of their gambling behavior, (iii) had reached the maximum deposit limit, (iv) had set a personal deposit limit because they had to, (v) had set a personal deposit limit to better control their gambling expenditure, and (vi) did not know why they set a personal deposit limit.

Self-reported problem gambling was negatively correlated with age (i.e., younger players had a higher likelihood of being a self-reported problem gambler). Players who registered more recently with the gambling operator were also more likely to be problem gamblers. Table 3 shows that self-reported problem gambling was not related to actual gambling behavior 30 days prior to answering the survey. There was no correlation between self-reported problem gambling and depositing money, wagering money, withdrawing money, or any other of the account-based player tracking metrics.

**Table 3:** Logistic regression with self-reported problem gambling as dependent variable and survey answers as well as actual gambling behavior of the past 30 days as independent variables

	Coefficient	Std.Err.	Z	*p*>|Z|	Sig.
I feel positive towards the €1000 maximum deposit limit	0.03	0.08	0.40	0.6928	
I believe that a maximum deposit limit is relevant for me	0.30	0.08	3.95	0.0001	*
I believe that generally I have a sufficient overview of my gambling expenditure	− 0.40	0.09	− 4.57	< 0.0001	*
Deposit limits help me to maintain a sufficient overview of, and control over, how much money I lose	0.08	0.09	0.90	0.3707	
I have never reached the maximum deposit limit	− 0.51	0.26	− 2.00	0.046	*
I did not play for the rest of the month	− 0.31	0.28	− 1.09	0.2746	
I did not play until I was permitted again	− 0.06	0.26	− 0.23	0.8177	
I continued to play with other compliant operators	0.19	0.33	0.57	0.5688	
I continued to play with other non-compliant online operators	0.41	0.35	1.14	0.2525	
I continued to play with land-based operators	0.64	0.49	1.32	0.1869	
I set a personal deposit limit because I have to	1.50	0.31	4.80	< 0.0001	*
I set a personal deposit limit to better control my gambling expenditure	0.88	0.32	2.77	0.0056	*
I don't know why I set a deposit limit	0.72	0.33	2.21	0.027	*
Female	0.23	0.28	0.80	0.4224	
Age	− 0.02	0.01	− 2.75	0.0059	*
Number of months since registration	− 0.16	0.04	− 4.16	< 0.0001	*
Amount deposited	0.00	0.00	− 1.46	0.144	
Amount wagered	0.00	0.00	1.15	0.2484	
Amount lost	0.00	0.00	0.21	0.8325	
Number of deposits	0.01	0.02	0.97	0.3341	
Number of withdrawals	0.05	0.04	1.23	0.2179	
Number of wagers	0.00	0.00	− 1.23	0.2173	
Number of gambling days	0.03	0.03	0.87	0.3824	
Average amount deposited	0.00	0.01	0.59	0.5559	
Average amount wagered	− 0.08	0.81	− 0.10	0.9185	
Standard deviation amount wagered	− 1.25	1.41	− 0.89	0.3742	
Number of different games played	0.01	0.01	0.57	0.5673	

## Discussion

In the present study, 1000 players at a German online slot game operator answered an online survey about their attitude towards a maximum monthly deposit limit and deposit limits in general. Players also answered the three questions of the Brief Biosocial Gambling Screen (Brett et al., 2014). These data were then combined with a second dataset comprising the players’ account-based actual gambling behavior. The average age of the gamblers was 40 years and 18% were female. Over half of the survey responses (58.7%) were completed by players who had been registered with the operator for at least six months which suggests that they had experience with the operator’s gambling products.

Asked whether they felt positive towards the €1000 monthly maximum deposit limit 62.4% of players agreed in part or entirely. In their survey of Norwegian online players, Auer, et al. (2020) reported that 79% of players agreed in part or entirely when asked whether they felt positive towards a NOK 20,000 monthly loss limit (approximately $2000 US). However, the two studies were based on players from different countries with different regulations. Norway has an online gambling monopoly whereas Germany has a licensed online gambling market. Furthermore, *Norsk Tipping*, the Norwegian monopoly operator, offers lottery games, table games, sports betting, and slots games. The present study’s online gambling operator only offers slots games. In an old survey of attitudes towards responsible gambling, Bernhard et al. (2006) found that problem gamblers and problem gamblers in recovery disliked systems that forced limits upon them, invoking concerns about ‘Big Brother’ watching over their play.

In the present survey, 36% of players agreed in part or entirely that a maximum deposit limit was relevant for them. The same percentage was reported by Auer et al. (2020). Two-thirds of players (65%) agreed entirely or in part when asked whether they believed they had a sufficient overview of their gambling expenditure. Auer et al. (2020) reported that 91% of players said they had a sufficient overview of their gambling. The lower percentage in the present study could be related to slots games’ structural characteristics. Slots games generally have a higher event frequency compared to table games or lottery games. When comparing self-reported losses with actual losses, Auer and Griffiths (2017) found that the accuracy of self-reported losses varied across game preferences. This could also have had an impact on players’ statements regarding the overview of their gambling.

Just under half of players (47.7%) agreed entirely or in part when asked whether deposit limits were helpful in maintaining a sufficient overview of, and control over, how much money they lost. Auer et al. (2020) reported a similar percentage (45% of players) saying loss limits helped them to maintain a sufficient overview of, and control over, how much money they lose. Three-fifths of players (60.5%) said that they had reached the maximum monthly deposit limit at least once. However, in reality only 7.6% of players deposited €1000 in at least 1 month since they registered with the gambling operator. This result could be related to the fact that players also had to choose a personal monthly deposit limit which of course had to be lower than €1000. It is probably the case that players did not distinguish between reaching their own personal monthly deposit limit and the legal maximum monthly deposit limit when answering the survey question.

It was also mandatory for players to set a monthly deposit limit at registration which has to be lower than €1000. A limit increase only comes into effect on the first day of the following month. Therefore, players may have answered this particular question based on players reaching their personal deposit limit rather than the €1000 limit. The low percentage of players who actually ever deposit the maximum amount is line with a study by Broda et al. (2008) who found that only a minority of players tried to exceed their personal deposit limit. Of the 76 players who deposited €1000 in at least 1 month, 72 of them were within the 605 players who also self-reported they did so. This means that players who actually reached the monthly €1000 maximum deposit limit also said so which increases the validity of the results.

Out of the 605 players who said that they had reached the maximum monthly deposit limit, 42% said that they then continued to gamble elsewhere. In the study by Auer et al. (2020) only 10% of players said they continued to gamble elsewhere after reaching their monthly deposit limit. As aforementioned, Norway has an online gambling monopoly and at the time of the present survey, players could continue to play with any other German online gambling operator. Also, Auer et al. (2020) did not have access to the account-based data of those who actually did reach their limit, whereas in the present study only 76 players actually did. However, 605 players claimed that they did.

Players who said they reached the monthly deposit limit in the survey wagered and deposited more money in the 30 days prior to completing the survey compared to players who said they did not reach the limit. The same holds true for players who actually reached the monthly deposit limit at least once in the tracking data. This shows that the self-reported data correlated with actual gambling behavior, although only a minority of those who said they reached the monthly deposit limit in the survey actually did so when compared to their tracking data. Auer et al. (2020) assessed gambling intensity using the gambler’s *PlayScan* status. *PlayScan* is a behavioral tracking tool which classifies players into ‘green’ (low-risk), ‘yellow’ (medium-risk), and ‘red’ (high-risk) based on actual gambling behavior. Auer et al. (2020) also found that high-risk players were less favorable towards the maximum monthly monetary loss limit.

There was no significant different between players who agreed that the maximum deposit limit was relevant to themselves and players who disagreed with respect to amount of money wagered and deposited. There was also no significant difference in amount of money wagered and deposited regarding the questions about having a sufficient overview of gambling and the benefit of limits to maintain a sufficient overview. This could be due to the fact that players were not aware of their gambling expenditures. Previous studies have shown that players who gamble regularly tend to underestimate their losses and overestimate their winnings (Auer & Griffiths, 2017; Braverman et al. 2014).

The 605 players who said that they continued to gamble elsewhere after reaching their maximum limit, deposited and wagered more money in the 30 days prior to the survey compared to players who said that they did not continue to gamble elsewhere. The same pattern was found for the 76 players who actually deposited €1000 in at least one month. Players who said they chose a personal deposit limit because it helped them to better control their gambling expenditure, deposited and wagered more money than players who did not agree with that statement. This indicates that more gambling-intense players choose limits that take into account their gambling expenditure when choosing their limit.

Players also answered the three questions in the Brief Biosocial Gambling Screen. In total 21.2% endorsed one or more of the three questions. In a previous survey of online gamblers using the same screen, 27% of participants agreed to one or more of the three BBGS questions (Louderback et al., 2021). They were also able to predict self-reported problem gambling with player tracking data which reflected depositing, wagering, winning, and playing time.

In a logistic regression with self-reported problem gambling as the independent variable, none of the variables reflecting actual gambling behavior were significant. This means that self-reported problem gambling was not related to how players actually gambled at all. Self-reported problem gamblers did not deposit or wager more money than non-problem gamblers. This is in contrast to previous studies which have found correlations between self-reported problem gambling and actual gambling behavior (Louderback et al., 2021; Luquiens et al., 2016). One explanation could be that players are simply not aware of an underlying gambling problem. Several studies have shown that players are not aware of their gambling expenditure (Auer & Griffiths, 2017; Braverman et al., 2014). It should also be noted that overall expenditure in and of itself is not a good indicator of problem gambling given the diversity of what gamblers can afford to lose.

Another explanation could be that players did not answer the question honestly, fearing that the results could be misused. Bernhard et al. (2006) reported that players mentioned concerns about being monitored in relation to responsible gambling procedures. However, there was an association between self-reported problem gambling and several other survey responses. Players who did not agree that they had a sufficient overview of their gambling were more likely to be problem gamblers. Players who said that a maximum limit was relevant to them were also more likely to be problem gamblers. A higher percentage of problem gamblers was also observed among players who said that they continued to gamble elsewhere after reaching the maximum monthly deposit limit. Those correlations between self-reported problem gambling and survey responses appear logical and valid.

There was also a negative association between problem gambling and age. In their analysis of online poker players, Luquiens et al. (2016) also found problem gamblers to be younger than non-problem gamblers. Players who registered more recently were also more likely to self-report problem gambling. Problem gamblers might also simply be drawn to other platforms without a maximum deposit limit and only play occasionally with a regulated operator. The self-reported problem gamblers might also already have reduced their gambling after experiencing the negative impacts of gambling. Players who reported as having problems based on the Brief Biosocial Gambling Screen did not deposit more money or spend more money than non-problem gamblers when examining their account-based tracking data. However, gambling expenditure on its own is not necessarily a good proxy for problem gambling especially as the present authors had no idea of what disposable income the participants had. For those on poor incomes, even losing very small amounts of money may be problematic.

## Limitations

The present study has a number of limitations. Although the study combined subjective self-report data with objective account-based data, both types of data have methodological shortcomings. All self-report data are open to biases (e.g., memory recall, social desirability) and the some of the findings showed that what players thought they had done in relation to some of their gambling behavior did not always equate to what they had actually done. The survey data were also cross-sectional (limiting any determination of causality between the study variables) whereas the account-based data had a longitudinal dimension. Also, the four items used to assess participants’ attitudes toward deposit limits are arguably more suggestive than objective (e.g., *“I feel positive towards the €1000 maximum deposit limit”* answered from ‘Disagree entirely’ to ‘Agree entirely’). A differently-worded question such as *“What is your attitude toward the deposit limit?”* assessed on a seven-point Likert scale from 1 (*very negative*) to 7 (*very positive*) might have produced different results. However, the present authors had no role in either the design or implementation of the survey and simply analysed a dataset provided by the gambling operator.

It should also be noted that the sample size in the present survey was very modest compared to most previously published behavioral tracking studies although for survey studies the sample size is quite large. However, the sample was not necessarily representative of online gamblers given that the operator only has one type of gambling game in its portfolio (i.e., online slot machine games) and all the gamblers were presumably German (and at the very least German-speaking) so there may also be cultural differences if the study was replicated with online gamblers from other countries. There is also a small possibility that some of the gambling accounts (in relation to the account-based tracking data) may have been used by more than one individual (e.g., married couples) although the number of shared accounts is likely to be small.

## Conclusion

The German State Treaty on gambling now limits the maximum amount of money deposited per month to €1000. At the time of writing, these players could deposit €1000 with several compliant operators. In the future, the €1000 limit will be across all operators not per operator. A maximum spend limit should protect many players from experiencing financial harm through gambling. However, players can still migrate to non-German gambling operators to circumvent the maximum deposit limit. For this reason, it is important to gain more insights into attitudes towards a maximum deposit limit. Generally, players in the present study were in favor of the maximum deposit limit. However, players who deposited and wagered more money were less favorable towards the measure. Moreover, 42% of the players who said they had reached their maximum deposit limit said that they carried on gambling elsewhere. This would have a negative impact on channelization of gambling into the German regulated market and also undermine the player protection efforts of the German regulator. The fact that players who said they carried on gambling after reaching the limit deposited and wagered more money in the 30 days prior to completing the survey is another reason why further research into the impact of maximum deposit limits is necessary. This is further underlined by the finding that players who disagreed with the maximum deposit limit deposited more money in the 30 days prior to completing the survey. This group of players are the more high-intensity players and it is this group of players that regulation is often trying to protect the most. However, the gambling regulators in Germany need to think about the possible unintended consequences of problem gamblers simply playing with non-German and potentially less socially responsible operators to avoid limits that they perceive as too restrictive.

## Data Availability

The two datasets used for this study were supplied by an online gambling operator and are both commercially sensitive. Therefore, the datasets are not available to other researchers on this occasion.
